# Ulzerierte Unterlidschwellung bei einem Säugling

**DOI:** 10.1007/s00347-020-01098-2

**Published:** 2020-04-14

**Authors:** Helena Wagner, Julia Biermann, Wolf Alexander Lagrèze, Claudia Auw-Hädrich

**Affiliations:** 1 Klinik für Augenheilkunde, Universitätsklinikum Freiburg, Killianstr. 5, 79106 Freiburg, Deutschland; 2 Medizinische Fakultät, Albert-Ludwigs-Universität Freiburg, Freiburg, Deutschland; 3 Klinik für Augenheilkunde, Universitätsklinikum Münster, Münster, Deutschland

## Anamnese

**Figure 1:**
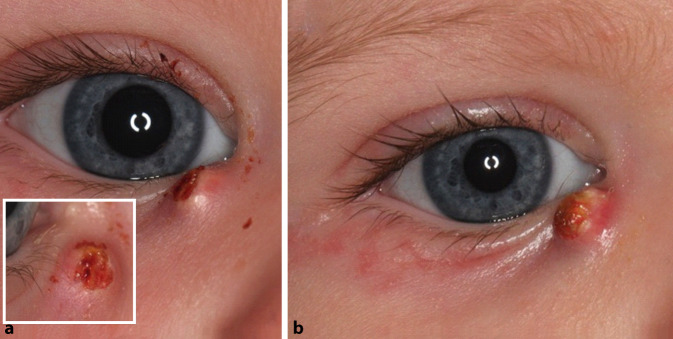

Ein 10 Monate altes gesundes Mädchen zeigte eine schnell wachsende Raumforderung im Bereich des medialen Unterlids (Abb. 1a). Diese Veränderung wurde erstmals 10 Wochen vor der Vorstellung in unserer Klinik nach einem vermuteten Insektenstich beobachtet. Zuvor hatte die Patientin im Alter von 2 Monaten eine Tränenwegsstenose gezeigt, die spontan rückläufig war.

Anamnestisch wurden vom niedergelassenen Augenarzt bereits topische und systemische Antibiotika verordnet, auf die die Schwellung nicht reagierte. Die Mutter berichtete über eine große Variation des Tumoraussehens (unterschiedliche Größe, Abfallen der Oberflächenkruste, Perforation, spontane Blutung; Abb. 1b). Es lagen weder Epiphora, Juckreiz noch Schmerzen vor.

## Befund

Wir beobachteten eine scharf abgegrenzte, sich vorwölbende, teils mit rötlichen, teils mit gelblichen Krusten belegte Raumforderung unterhalb des rechten medialen Lidwinkels. Der weitere ophthalmologische Befund war unauffällig. Bei Persistenz der Raumforderung trotz Antibiotikatherapie entschieden wir uns daher zur vollständigen exzisionalen Biopsie der oben beschriebenen Läsion unter Vollnarkose.

## Exzision

Intraoperativ zeigte sich der Tumor mit zystischen Formationen und weißlichen, granulomatös anmutenden Elementen, ohne Verbindung zum Tränenwegssystem. Der primäre Wundverschluss wurde mit resorbierbaren Nähten durchgeführt. Die Heilung verlief ohne Komplikationen.

## Histologie

**Figure 2:**
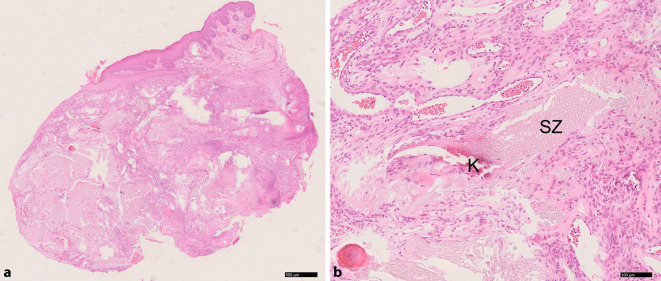

Die Hämatoxylin-Eosin-Färbung der Paraffinschnitte zeigte verschiedene Inseln kernfreier Schattenzellen im tieferen subkutanen Gewebe und oberflächlich im ulzerierten Bereich. Zudem stellten sich eine basophile Zellproliferation mit Kalziumablagerung im umgebenden Gewebe mit begleitender riesenzellhaltiger granulomatöser Entzündung und kapillarenhaltiges Granulationsgewebe dar (Abb. 2).

## Wie lautet Ihre Diagnose?

**Diagnose:** Perforierendes Pilomatrixom

Aufgrund dieser Befunde wurde die Diagnose des perforierenden Pilomatrixoms gestellt.

## Diskussion

### Definition

Das Pilomatrixom ist eine seltene gutartige Neubildung der Haut, die vom Haarfollikel ausgeht. Es ist ein relativ seltener Tumor mit einer je nach Literaturquelle stark variablen Inzidenz. In einer Studie mit 205 Pilomatrixom-Fällen lag die Inzidenz bei 1,04 % aller gutartigen Hauttumoren [1], während andere Autoren niedrigere Werte von 0,001–0,0031 % aller dermatologischen histologischen Proben fanden [2]. Es tritt normalerweise im Kopf- und Nackenbereich von Kindern und Jugendlichen auf, kann jedoch überall im Körper und auch bei älteren Menschen beobachtet werden. In den Fallserien wurde das periokulare Pilomatrixom meistens als langsam wachsender asymptomatischer Knoten mit normaler, darüber liegender Haut dokumentiert [4]. Gelegentlich wurden rötliche bis blaue Verfärbungen der Haut oder ein gestieltes Wachstum beschrieben [4], häufig mit histologisch nachweisbarer Verkalkung. Eine seltene klinische Variante ist das perforierende Pilomatrixom, das als verkrusteter Knoten oder ulzerierter Tumor auftritt und schneller als das klassische Pilomatrixom wächst. Somit könnte ein Pilomatrixom andere häufigere Augenlidtumoren imitieren, z. B. Epidermiszyste, pyogenes Granulom, Keratoakanthom.

### Diagnostik und Histologie

Die korrekte Diagnose kann erst nach Exzision und histologischer Untersuchung gestellt werden. Charakteristisch sind das histopathologische Nebeneinander von Schattenzellen, Basaloidzellen sowie eine Entzündungsreaktion, die von riesigen mehrkernigen Zellen und Verkalkungen dominiert wird. Schattenzellen an sich sind für Pilomatrixome nicht pathognomonisch, da sie selten auch bei anderen Hauttumoren auftreten können. Ein reiches Netzwerk von Blutgefäßen mit einer hohen Gefäßdichte, wie in unserem Fall berichtet, wurde bereits von Indrei beschrieben [3].

### Lokalisation und Erscheinung

Die am häufigsten betroffenen periokularen Lokalisationen sind das obere Augenlid oder die Augenbraue. Das Unterlid oder der mediale Kanthus sind selten einbezogen. Nach unserem Kenntnisstand wurde bislang noch nie das Auftreten eines klassischen Pilomatrixoms vor dem Erreichen des ersten Lebensjahres beschrieben, und ein perforierendes Pilomatrixom wurde bislang noch nicht bei Patienten unter 11 Jahren beobachtet. Über periokuläre perforierende Pilomatrixome bei älteren Erwachsenen wurde in der Literatur selten berichtet. Darüber hinaus war das klinische Erscheinungsbild des Säuglings aufgrund der Lage des Tumors mit sich ändernder Größe, Oberflächenkruste und spontanen Blutungen recht ungewöhnlich. Letztere sind durch das gefäßreiche Granulationsgewebe zwischen den Epithelinseln erklärt.

### Therapie

Aufgrund der Lage des Tumors und des rötlichen Aussehens sowie des Alters der Patientin schien eine perforierte Dakryozystitis bzw. ein pyogenes Granulom eine wichtige Differenzialdiagnose zu sein. Das Fehlen von Epiphora oder allgemeinem Unwohlsein und die Persistenz trotz Antibiotikatherapie sprachen jedoch gegen eine Tränenwegsstenose bzw. ein infektiöses Geschehen. Da laut Literatur keine spontane Regression von Pilomatrixomen beobachtet wurde, sollte eine vollständige Exzision angestrebt werden [5]. Darüber hinaus ist das Wiederauftreten eines Pilomatrixoms selten [1], was eine Operation mit geringem Sicherheitsabstand wie in unserem Fall rechtfertigt. Bei einer Lokalisation des Pilomatrixoms in anderen Körperregionen oder bei älteren Patienten kann zur Sicherung der vollständigen Exzision ggf. ein größerer Sicherheitsabstand gewählt werden als im dargestellten Fall.

## Fazit für die Praxis

- Das Pilomatrixom ist meist ein gutartiger Hauttumor, der aus Haarfollikelmatrixzellen stammt und in der Regel als asymptomatischer, langsam wachsender Knoten auftritt.
- Eine seltene Variante ist das perforierende Pilomatrixom, ein schnell wachsender verkrusteter oder ulzerierter Tumor.
- Periokular sind das Oberlid oder die Augenbraue am häufigsten betroffen.
- Wenn der Bereich unterhalb des medialen Lidwinkels betroffen ist, kann das perforierende Pilomatrixom eine Dakryozystitis vortäuschen.
